# Nasopalatine duct cyst with sebaceous differentiation: a rare case report with literature review

**DOI:** 10.1186/s12903-021-01772-0

**Published:** 2021-08-26

**Authors:** Han-Gyeol Yeom, Jae-Hyun Kang, Sun-Ung Yun, Jung-Hoon Yoon

**Affiliations:** 1 Department of Oral and Maxillofacial Radiology, Daejeon Dental Hospital, Wonkwang University College of Dentistry, Daejeon, Korea; 2 Department of Oral and Maxillofacial Surgery, Daejeon Dental Hospital, Wonkwang University College of Dentistry, Daejeon, Korea; 3 Department of Oral and Maxillofacial Surgery, Yuseong Sun Dental Hospital, Daejeon, Korea; 4 Department of Oral and Maxillofacial Pathology, Daejeon Dental Hospital, Wonkwang University College of Dentistry, Daejeon, Korea

**Keywords:** Nasopalatine duct cyst, Incisive canal cyst, Sebaceous differentiation

## Abstract

**Background:**

The aim of this study was to report a rare case of nasopalatine duct cyst with sebaceous differentiation. Further, a systematic search of the literature was performed to identify studies reporting patients with intraosseous jaw cysts with sebaceous differentiation.

**Case presentation:**

A 55-year-old Korean man was referred to our hospital because of a cystic lesion of the anterior maxilla. Radiologic examination revealed a well-circumscribed radiolucent lesion in the anterior maxilla. Histology showed a respiratory columnar and cuboidal epithelium-lined cyst. Transition from the ciliated columnar epithelium to stratified squamous epithelium with sebaceous differentiation was observed. Based on these findings, the final diagnosis was nasopalatine duct cyst with sebaceous differentiation. A systematic search of the literature was performed to identify studies reporting patients with intraosseous jaw cysts with sebaceous differentiation. There were 24 cases of sebaceous differentiation in the epithelium of the cysts including 2 odontogenic keratocysts, 8 orthokeratinized odontogenic cysts, 8 dentigerous cysts, 1 radicular cyst, and 2 glandular odontogenic cysts. However, no case reports describing the occurrence of nasopalatine duct cysts with sebaceous differentiation have been reported.

**Conclusion:**

This first case report of nasopalatine duct cysts with sebaceous differentiation could provide insight into the diagnostic process of cystic lesions with sebaceous differentiation.

## Background

Nasopalatine duct cysts (NPDCs), also known as incisive canal cysts, are the most common non-odontogenic developmental cysts in the jaws [1, 2]. As the lesions are usually asymptomatic, NPDC is discovered mostly on routine panoramic radiographs [2]. Histologically, squamous, ciliated (respiratory), and cuboidal epithelium are found in these cysts. More than one epithelial type is commonly observed, and the type of epithelium depends on the location involved (palatine, nasal, or intermediate) [1, 2]. The etiology and pathogenesis of these cysts are unknown, but some investigators have proposed that NPDCs develop from the spontaneous proliferation of the remnants of embryonic tissue [1–4]. Epithelial remnants of the nasopalatine duct may be stimulated to proliferate by trauma, infection, or mucous retention [1, 2, 4]. As the cysts have been found in human fetal incisive canals, spontaneous cystic degeneration of epithelial remnants is also suggested [1, 2].

Sebaceous glands are prominent skin appendage components formed in close association with or independent of hair follicles [5, 6]. Sebaceous glands are generally found in most parts of the body [6] and are very common in the oral mucosa [5, 6]. Within the oral cavity, sebaceous glands may present as small, yellowish spots called Fordyce granules, which exhibit a predilection for the buccal mucosa [7–9].

Some cases of intraosseous jaw cysts with sebaceous differentiation have been reported [10–16]. There were 24 cases of sebaceous differentiation in the epithelium of the cysts including odontogenic keratocysts (OKCs), orthokeratinized odontogenic cysts (OOCs), dentigerous cysts (DCs), radicular cysts (RCs), and glandular odontogenic cysts (GOCs). However, we found no case report describing the occurrence of NPDC with sebaceous differentiation in our search of the English literature. Here, we report a rare case of NPDC with sebaceous differentiation. In addition, a systematic search of the literature was performed to identify studies reporting patients with intraosseous jaw cysts with sebaceous differentiation.

## Case presentation

A 55-year-old Korean man was referred to our hospital from a local dental clinic because of a cystic lesion in the anterior maxilla. The patient had no pain or significant systemic disease.

**Fig. 1:**
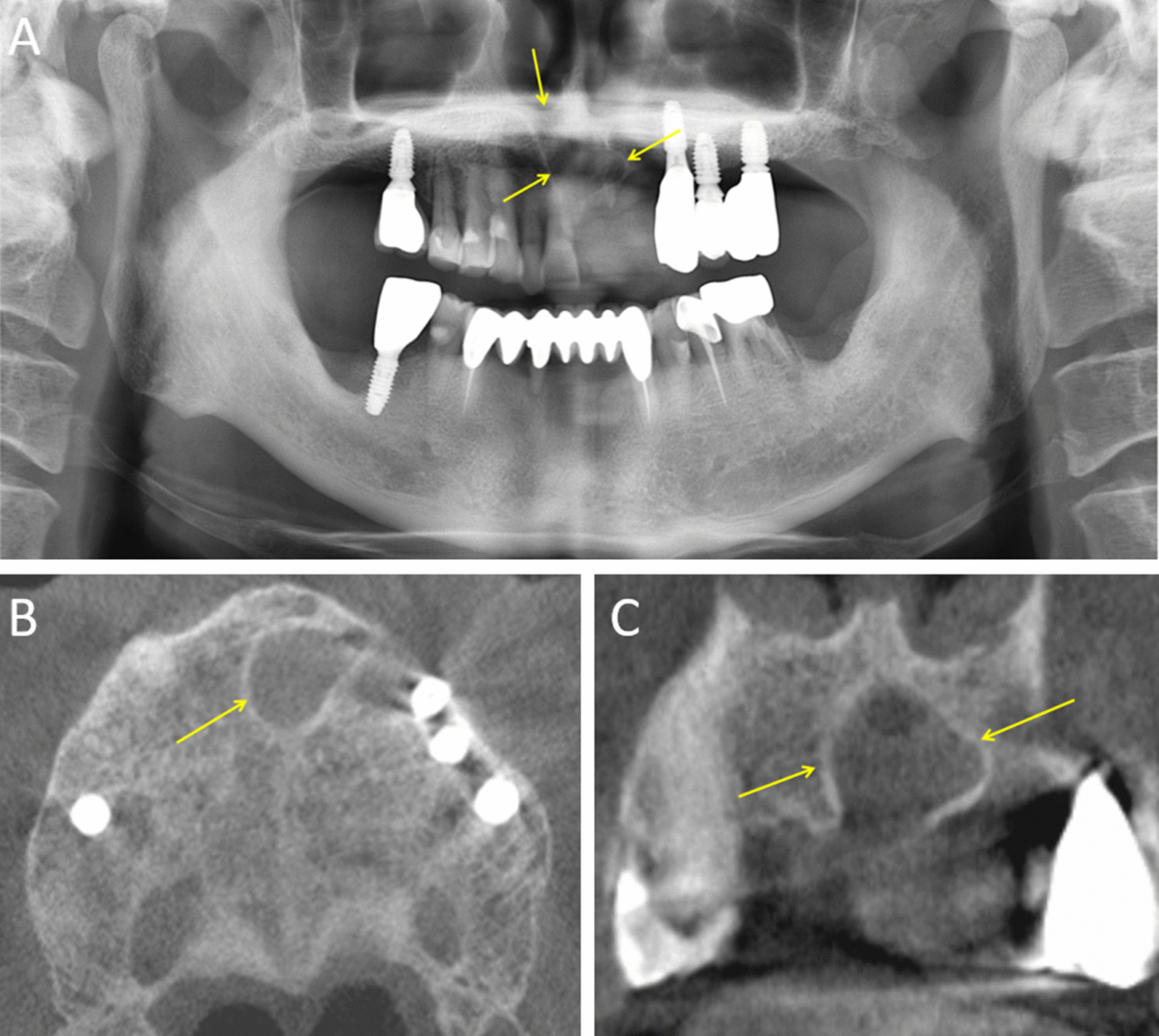
**a** Panoramic radiograph showing a radiolucent lesion in the anterior maxilla (yellow arrow). **b**, **c** Cone-beam computed tomography scan showing a well-defined corticated cystic lesion. (B. axial, C. coronal)

Clinical examination revealed no distinct expansion of the anterior maxilla. Left maxillary incisors were lost. Panoramic radiography revealed a well-circumscribed radiolucent lesion in the anterior maxilla (Fig. 1a). The border of the lesion was well-defined, with a corticated margin. No apparent external root resorption of the adjacent teeth was observed, and no normal nasopalatine canal structures were observed.

Additional cone-beam computed tomography scans revealed a well-defined corticated lesion. As the lesion was not large enough to affect the labial or palatal cortical bone, no apparent expansion pattern of the labial and palatal cortex was observed (Fig. 1b, c).

An ovoid-shaped, low-attenuated lesion was observed in the anterior part of the maxilla (yellow arrow). Neither an apparent expansion pattern nor deviation of adjacent structures was observed. Radiological and clinical diagnosis of NPDC was made.

**Fig. 2:**
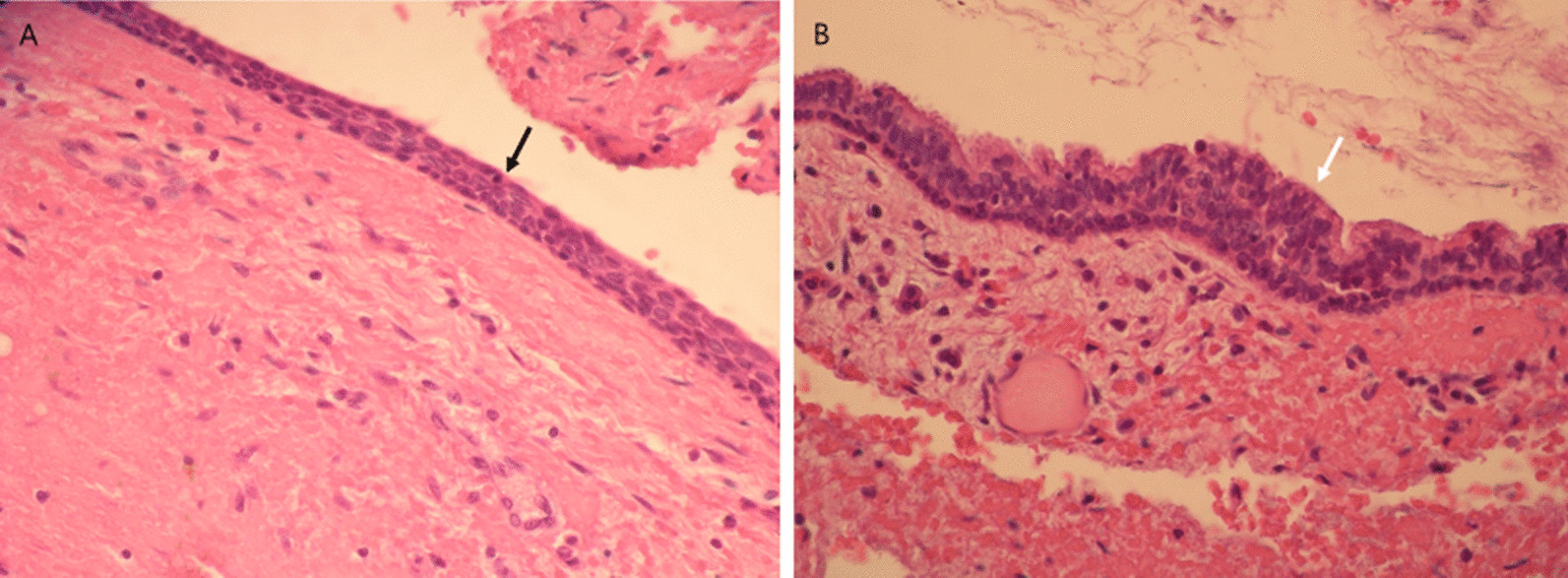
**a**, **b** The cyst is lined by cuboidal epithelium (A, black arrow) and ciliated columnar epithelium (B, white arrow).

**Fig. 3:**
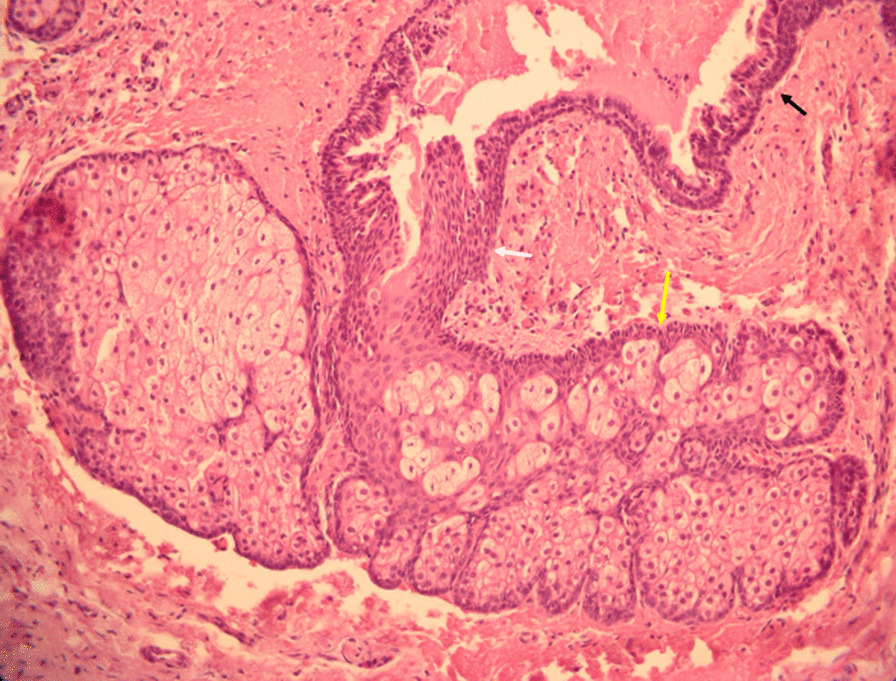
The lining epithelium showed a transition from the ciliated columnar epithelium (black arrow) to stratified squamous epithelium (white arrow) with sebaceous differentiation (yellow arrow)

Surgical cyst enucleation and histopathological examination were performed. Histopathologically, the lesion consisted of a cuboidal and respiratory ciliated columnar epithelium-lined cyst (Fig. 2a,b). Transition from the ciliated columnar epithelium to stratified squamous epithelium with sebaceous differentiation was observed (Fig. 3). Considering both histopathological and radiographic properties, the final diagnosis made was NPDC with sebaceous differentiation.

## Discussion and conclusion

A systematic search of the literature was performed to identify studies reporting patients with intraosseous jaw cysts with sebaceous differentiation. The PubMed/MEDLINE/Google Scholar databases and gray literature were searched for English language papers using a combination of terms such as “intraosseous” or “jaw” or “maxilla (maxillary)” or “mandible (mandibular),” “cyst” or “cystic lesion” and “sebaceous.” The literature from 1980 to 2020 was searched. Papers that allowed access only to the abstract were excluded.

As a result, a total of 88 papers were reviewed, and finally, seven papers, including case series, case reports, and articles investigating the imaging and histopathologic appearance of cystic lesions [10–16], with 24 patients were selected. Table 1 summarizes the data obtained from the selected papers.

**Table 1:** Clinicopathologic characteristics of patients have odontogenic cysts with sebaceous glands

Authors	Year	Sample size	Age (years)	Sex	Location	Radiologic features	Symptom	Treatment	Final diagnosis
RE Christensen Jr et al. [10]	1982	1	20	F	Left posterior mandible	Unilocular radiolucency in the left mandibular third molar area	No pain or numbness, discomfort	N/A	OKC
E Vuhahula et al. [11]	1993	1^*^	21	M	Right anterior maxilla	Unilocular radiolucency radiologically diagnosed periapical cyst	N/A	Enucleation	OOC^†^
AC Chi et al. [12]	2007	5	44	F	Left posterior mandible associated with impacted third molar	Well-circumscribed radiolucency of approximately 2.5 cm in maximum diameter associated with the crown of an impacted left mandibular third molar	No pain, no discomfort	Tooth extraction and cyst enucleation	OOC
			28	F	Left posterior mandible	Well-circumscribed radiolucency of approximately 5.5 cm in maximum diameter surrounding the crown	Asymptomatic	Tooth extraction and cyst enucleation	OOC
			20	M	Left maxillary sinus associated with impacted left maxillary third molar	Well-delineated, partially corticated radiolucency in the area of the left maxillary sinus	Asymptomatic	Cyst enucleation	OOC
			24	M	Left posterior mandible associated with impacted third molar	Well-circumscribed radiolucency associated with an impacted right mandibular third molar	Asymptomatic	Tooth extraction and cyst enucleation	OOC
			13	M	Left posterior mandible	Well-circumscribed radiolucency associated with the crown of an unerupted left mandibular premolar	No pain, no discomfort	Cyst enucleation	DC^§^
T Shamim et al. [13]	2008	1	12	F	Right posterior mandible	Biloculated radiolucency on right posterior mandible	Swelling	Surgical exploration	OKC
M Kumar et al. [14]	2014	1	18	M	Anterior maxilla	Unilocular, ovoid-well-defined radiolucency	Pain	Apicoectomy	RC
APN Aksakallı [15]	2018	14	25	F	Right posterior mandible	Multilocular, well-defined radiolucency	N/A	Enucleation	OKC
			59	M	Right posterior mandible	Unilocular, ovoid-well-defined radiolucency	N/A	Enucleation	OKC
			23	M	Left posterior mandible	Unilocular, ovoid-well-defined radiolucency	N/A	Enucleation	OOC
			14	F	Right first molar-left canine region	Unilocular, ovoid-well-defined radiolucency	N/A	Enucleation	OOC
			31	M	Left anterior maxilla associated with impacted canine	Unilocular, ovoid-well-defined radiolucency	N/A	Enucleation	DC
			54	F	Right anterior maxilla associated with impacted canine	Unilocular, ovoid-well-defined radiolucency	N/A	Enucleation	DC
			53	M	right first molar-left second premolar region, associated with impacted left canine	Unilocular, ovoid-well-defined mixed radiolucency	N/A	Enucleation	DC + compound odontoma
			55	M	right posterior mandible associated with impacted third molar	Unilocular, scalloped radiolucency	N/A	Incisional biopsy (marsupialization)	GOC
			27	F	left anterior maxilla associated with impacted canine	Unilocular, ovoid-well-defined radiolucency	N/A	Enucleation	DC
			73	F	RIGHT anterior mandible	Unilocular, scalloped radiolucency	N/A	Enucleation	OKC
			21	M	Right posterior mandible associated with impacted third molar	unilocular, ovoid-well-defined radiolucency	N/A	Enucleation	DC
			50	M	Right posterior mandible	Multilocular, well-defined radiolucency	N/A	Enucleation	GOC
			60	M	Right anterior maxilla associated with impacted canine	Unilocular, ovoid-well-defined radiolucency	N/A	Incisional biopsy	DC
			42	M	Left posterior mandible associated with impacted third molar	Unilocular, ovoid-well-defined radiolucency	N/A	Enucleation	DC
L Kavitha et al. [16]	2020	1	24	M	Left posterior mandible	Unilocular, scalloped well-defined radiolucency	Mild pain	Excision	OOC

OKC: odontogenic keratocyst, OOC: orthokeratinized odontogenic cyst, DC: dentigerous cyst, RC: radicular cyst, GOC: glandular odontogenic cyst, N/A: not available

*Case no. 11 was used

^†^The term of “Orthokeratinized jaw cyst” was used

^§^Reclassifying as OOC was on review

There were 24 cases of sebaceous differentiation in the epithelium of the cysts, but no case of sebaceous differentiation in NPDC was reported. Out of the 24 cases, there were 8 (33%) cases of OOC and DC, respectively, 5(21%) cases of OKC, 2(8%) cases of GOC, and 1(4%) case of RC. Sebaceous elements in the cystic epithelium probably represent sebaceous metaplasia [11]. Various hypotheses have been suggested for the etiopathogenesis. One hypothesis is that the origin might be the sequestered multipotent epithelial cells that aid in the development of the oral cavity, which may have been embedded deep in the surface and entrapped in the developing jaw during embryogenesis. Hence, its proliferation might have been induced by dental inflammation, trauma, or cystic change [15, 17, 18]. Another hypothesis is that cysts develop from the existing or cystic epithelium that undergoes dermal metaplasia [15, 17, 18]. Such theories can also be used to understand the sebaceous differentiation of NPDCs.

We proposed three possibilities for the etiology of this case. First, because of the chronic periodontitis or unknown traumas, sebaceous differentiation of entrapped multipotent epithelial cells occurred, and this was simply adjacent to the NPDC. Second, the NPDC occurred first, and subsequent adjacent multipotent epithelial cells were affected by stimuli of hydrostatic pressure due to the cystic change and underwent sebaceous differentiation. Third, NPDC occurred first followed by sebaceous metaplasia of the cyst lining epithelium.

All three hypotheses are possible, but considering the histopathologic features, the third hypothesis is thought to be the most likely. The sebaceous component was not simply adjacent to the NPDC but was connected along the cyst lining. In addition, a transition from the ciliated columnar epithelium to stratified squamous epithelium was observed in the epithelium where the sebaceous component was connected, so it is most likely that the sebaceous component also occurred among metaplasia in various directions.

In this paper, we described the first case of NPDC with sebaceous differentiation and suggest a possible etiology based on the results of a literature review conducted on the reports of sebaceous differentiation in various intraosseous jaw cysts. Although there are various limitations to the generalization of this study due to the small number of cases, we expect to improve the understanding and diagnosis of intraosseous jaw cysts with sebaceous differentiation by reporting this paper.

## Data Availability

All data analyzed during this study are included in this published article.

## Abbreviations

NPDC: Nasopalatine duct cyst
OKC: Odontogenic keratocyst
OOC: Orthokeratinized odontogenic cysts
DC: Dentigerous cyst
RC: Radicular cysts
GOC: Glandular odontogenic cyst
